# *N*-3-Methylbutyl-benzisoselenazol-3(2H)-one Exerts Antifungal Activity In Vitro and in a Mouse Model of Vulvovaginal Candidiasis

**DOI:** 10.3390/cimb46030157

**Published:** 2024-03-14

**Authors:** Xiuyi Liang, Agata J. Pacuła-Miszewska, Richa Vartak, Milankumar Prajapati, Haiyan Zheng, Caifeng Zhao, Ganming Mao, Ketankumar Patel, Natalya U. Fedosova, Jacek Ścianowski, Blase Billack

**Affiliations:** 1 Department of Pharmaceutical Sciences, St. John’s University, Queens, NY 11439, USA; 2 Faculty of Chemistry, Nicolaus Copernicus University, 87-100 Toruń, Poland; 3 Department of Pathology and Laboratory Medicine, Brown University, Providence, RI 02912, USA; milankumar_prajapati@brown.edu; 4 Center for Advanced Biotechnology and Medicine, Piscataway, NJ 08854, USA; 5 Department of Biomedicine, Aarhus University, 8000 Aarhus, Denmark; nf@biomed.au.dk

**Keywords:** *Candida albicans*, vulvovaginal candidiasis, ebselen analog, antifungal activity, Pma1p

## Abstract

In the present work, we evaluated the antifungal activities of two novel ebselen analogs, *N*-allyl-benzisoselenazol-3(2H)-one (*N*-allyl-bs) and *N*-3-methylbutylbenzisoselenazol-3(2H)-one (*N*-3mb-bs). Colorimetric and turbidity assays were performed to determine the minimum inhibitory concentration (MIC) of these compounds in S1 (fluconazole-sensitive) and S2 (fluconazole-resistant) strains of *C. albicans*. *N*-3mb-bs was more active than the *N*-allyl-bs compound. It is noteworthy that the concentration of *N*-3mb-bs observed to inhibit fungal growth by 50% (18.2 µM) was similar to the concentration observed to inhibit the activity of the yeast plasma membrane H^+^-ATPase (Pma1p) by 50% (19.6 µM). We next implemented a mouse model of vulvovaginal candidiasis (VVC) using the S1 strain and examined the mouse and yeast proteins present in the vaginal lavage fluid using proteomics. The yeast proteins detected were predominately glycolytic enzymes or virulence factors associated with *C. albicans* while the mouse proteins present in the lavage fluid included eosinophil peroxidase, desmocollin-1, and gasdermin-A. We then utilized the *N*-3mb-bs compound (12.5 mg/kg) in the mouse VVC model and observed that it significantly reduced the vaginal fungal burden, histopathological changes in vagina tissue, and expression of myeloperoxidase (MPO). All in all, the present work has identified a potentially promising drug candidate for VVC treatment.

## 1. Introduction

*Candida* spp. are opportunistic organisms that under certain circumstances can overgrow in body tissues such as the female reproductive tract and cause mucosal infections. Vulvovaginal candidiasis (VVC) is the most common opportunistic mucosal infection, affecting 75% of women, and the most common causative species are *C. albicans*, *C. glabrata*, and *C. tropicalis* [1,2,3]. *C. albicans* is a dimorphic fungus with different morphologies such as yeast and hyphae, the latter of which have importance in virulence. It is domicile on the skin, mucosa, and gastrointestinal tract of 30–50% of normal healthy individuals [4]. However, in some patients, *Candida* infections are recurrent and significantly affect quality of life [5]. The major symptoms of VVC include vaginal discharge, pruritus, dysuria, as well as dyspareunia and vaginal soreness [4,6].

Fluconazole (FLU) is an effective treatment for VVC. However, the emergence of drug-resistant *Candida* strains has been noted in immunocompromised settings, particularly in patients with advanced immunosuppression, as they are less sensitive to regular antifungal treatment [7,8,9]. Ebselen (EB) is a synthetic organoselenium compound that has been broadly studied in numerous in vitro and in vivo research studies. Due to its unique bioactivity, EB is included in clinical trials of various human disorders, both in the US and Asia [10]. EB is an effective inhibitor of the plasma membrane H^+^-ATPase proton pump (Pma1p), which is critical for fungal survival [11,12,13]. It has therefore proved to be a useful agent in experimental models of VVC [9,14,15,16].

**Figure 1:**
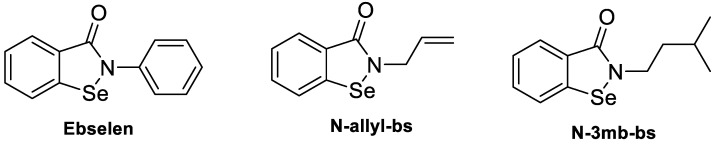
Chemical Structures of EB and its analogs *N*-allyl-bs and *N*-3mb-bs.

The toxicity of most selenium compounds limits their usefulness, but among them, EB is proposed to be a safe and effective treatment of mucocutaneous VVC when applied intravaginally [9]. However, due to its poor aqueous solubility, EB is difficult to formulate. As such a valuable compound, numerous analogs of EB have been synthesized to procure an EB analog with improved water solubility while maintaining its antifungal activity [16,17]. Here, we aimed to report the antifungal activities of two EB analogs, namely, *N*-allyl-benzisoselenazol-3(2H)-one (*N*-allyl-bs) and *N*-3-methylbutylbenzisoselenazol-3(2H)-one (*N*-3mb-bs), and to compare their antifungal activities to EB and the commercial products fluconazole (FLU) and miconazole both in vitro and in a mouse model of VVC. The chemical structures of these two analogs are depicted in Figure 1.

In addition, a proteomic analysis was carried out to confirm that the S1-induced VVC infection exhibited *C. albicans* virulence markers, and toxicity studies were also performed to evaluate the safety of the EB analogs.

## 2. Materials and Methods

### 2.1. Chemicals and Cells

The novel organoselenium compounds, *N*-allyl-bs and *N*-3mb-bs, were previously synthesized and described [18,19]. EB was purchased from AK Scientific, Inc. (cat# J54140, Union City, CA, USA). The *C. albicans* strains utilized in this research exhibit either sensitivity (clinical isolate S1) or resistance (clinical isolate S2) to FLU, with both strains generously provided by Dr. J. Morschhäuser from the University of Würzburg, Würzburg, Germany. Yeast extract–peptone–dextrose (YPD) was obtained from BD Diagnostic Systems in Sparks, MD, USA. FLU was procured from ApexBio (Cat #B2094) in Boston, MA, USA. Dimethylacetamide (DMA), Dimethyl sulfoxide (DMSO), Tween 80, estradiol valerate, and RPMI 1640 media were sourced from Sigma Aldrich in St. Louis, MO, USA.

MTT Reagent (3-[4,5-dimethylthiazol-2yl]-2,5-diphenyl-tetrazolium bromide) was purchased from Spectrum Chemical Mfg. Corp. (cat# MT1105GM, Gardena, CA, USA). KB-3-1 cell line was kindly provided by Dr. Zhe-Sheng Chen (St. John’s University, Jamaica, NY, USA). Miconazole cream (2%) was purchased from a local pharmacy (Clifton, NJ, USA). PBS and DMEM (Dulbecco’s Modified Eagle’s Medium) were purchased from Corning (Tewksbury, MA, USA).

### 2.2. Determination of Minimum Inhibitory Concentration (MIC)

The test compounds FLU, EB, *N*-allyl-bs, and *N*-3mb-bs were dissolved in DMSO in in vitro studies. For turbidity assays, *C. albicans* strains (S1 and S2) were cultured overnight in YPD medium. The *C. albicans* suspension was then adjusted to achieve an initial inoculum with an A600 of 0.010 in RPMI 1640 medium. The test compounds were serially diluted 1:2 in RPMI 1640 medium to create concentrations of 100, 50, 25, 12.5, 6.25, 3.12, 1.56, 0.78, and 0.39 μM. FLU was included as a positive control in the assay. In 24-well plates, each well received 200 μL of the yeast suspension followed by 200 μL of the appropriate test compound solutions. All plates were then incubated at 30 °C, and MIC values were recorded at both 24 and 48 h. A600 measurements were also taken to determine IC_50_ values. MIC is the lowest concentration of an antimicrobial drug that completely inhibits the visible growth of a microorganism after a specified incubation period. Due to its fungistatic nature, note that the MIC for FLU in the turbidity assay was defined as lowest concentration of FLU causing a 70% decrease in turbidity compared to the growth of control wells.

A colorimetric assay was conducted in triplicate for all test compounds using both S1 and S2 strains. The yeast suspension was adjusted to achieve an initial inoculum with an A600 of 0.010 in RPMI 1640 medium. In 96-well plates, each well was filled with 100 μL of the yeast suspension followed by 100 μL of the appropriate test compound solutions. All plates were then incubated at 30 °C. Following incubation, 20 μL of resazurin dye (0.02% *w*/*v*) was added to each well, and changes in dye color were observed to determine cell viability. MIC values were recorded at 24 and 48 h (MIC24h and MIC48h, respectively).

### 2.3. Medium Acidification Assay

A medium acidification assay was conducted to assess the inhibitory effects of the test compounds on the acidification of the growth medium by *C. albicans* S1 [11,13,15]. FLU was utilized as a negative control in the assay, as it was anticipated to have no impact on the plasma membrane H^+^-pump (Pma1p), which contributes to the acidification process. The concentration of test compounds necessary to inhibit medium acidification by 50% (IC_50MA_) was then determined based on a plot correlating the change in pH at 30 min with the concentration of the test compound, and these results were compared with those for untreated cells. All predicted log *p* values were obtained using the milogP calculator provided by the Molinspiration Property Calculation Service (www.molinspiration.com, accessed on 20 December 2023).

### 2.4. Cell Viability Assay

The cell viability assay was based on the method of Mosmann [20]. Briefly, 5 × 10^3^ of human epidermoid carcinoma cells KB-3-1 were seeded into each well in 96-well plates. The next day, the cells were treated with a series of concentrations of test compounds or the positive control FLU. After 24 h of treatment, 20 µL of 5 mg/mL MTT solution was added to each well and incubated for 3.5 h. DMSO was added to each well after the removal of MTT solution. The OD570 nm values were measured with an accuSkanTM GO UV/VIS Microplate Spectrophotometer (Fisher Sci., Fair Lawn, NJ, USA).

### 2.5. Validation of the Mouse Model of VVC and Intervention Study

The mouse model used in this study has been previously described by Yano and Fidel [21]. In brief, female BALB/c mice weighing between 18 and 22 g were procured from Taconic Laboratories, Inc. (Albany, NY, USA). These mice were housed in an Animal Care Center (ACC) at St. John’s University (Jamaica, NY, USA), which is accredited by the Association for Assessment and Accreditation of Laboratory Animal Care (AAALAC). This study was approved by the Institutional Animal Care and Use Committee (IACUC) of St. John’s University (Protocol #2003). The mice underwent a one-week acclimatization period in the ACC prior to the commencement of this study. The murine VVC study accounted for a total of 10 days (Supplementary Materials Figure S1). A total of 0.2 mg of β-estradiol valerate was dissolved in 100 µL sesame oil and subcutaneously injected on day −3 and on day 3 in order to ensure estrogen levels sufficient for the maintenance of the vaginal yeast infection. To establish a robust model of VVC, 20 µL (5 × 10^5^ CFU) of YPD containing *C. albicans* S1 was intravaginally introduced into the vaginal lumen on day 0, and the infection was allowed to progress until day 6.

#### 2.5.1. Proteomic Analysis of Vaginal Lavage Fluid

On day 6 post-infection, mice were euthanized and 100 µL of PBS was used to lavage the vagina. Lavage samples were then centrifuged at 10,000 rpm for 10 min at 4 °C to pellet any yeast in the lavage fluid. Next, 10 µL of supernatant containing yeast and mouse proteins but no cells was subjected to SDS-PAGE (100 V, 10 min). The gel was stained with Coomassie Blue overnight. The following day, bands were excised (4 mice for naïve and 4 mice for infected). Tryptic digests were analyzed using an Orbitrap Tribrid mass spectrometer and nanoflow LC system (Thermo Scientific, Waltham, MA, USA) as described in Barth et al. [22]. The raw liquid chromatography–mass spectrometry data were converted into MASCOT Generic Format using Proteome Discover 2.4 (Thermo Fisher, Waltham, MA, USA) and searched against either the Uniprot mouse proteome database or yeast database together with a database of common laboratory contaminants (https://www.thegpm.org/crap/, accessed on 12 December 2023) using a local implementation of the global proteome machine [23]. Raw counts were used to calculate the %abundance for each GPM-identified protein. Proteins with *p*-value < 0.05 and absolute log2 fold changes > 1 were called as differentially expressed for naïve control vs. infected lavage samples. A volcano plot was generated using GraphPad Prism (Version 10.1.1). Heatmaps on raw counts were generated using Morpheus at https://software.broadinstitute.org/morpheus/, accessed on 15 December 2023.

#### 2.5.2. Intervention Treatments

For intervention studies, mice were arranged into groups and either not treated and not infected (naïve) or estrogenized as described in 2.5 above on days −3 and +3 and inoculated with yeast on day 0. On days 3, 4, and 5 post-infection, a gel loading tip (highly flexible unlike sturdy pipette tips) was used for the intravaginal delivery of drugs. The tip was gently inserted not more than 5 mm deep into the vaginal lumen and the suspension of compounds injected. Animals were arranged into the following treatment groups (N = 6 mice per group):

Group 1: Naïve: non-infected (no additional treatments);

Group 2: 6th day infected: Estrogenized and infected (inoculated with yeast) with no additional treatments (baseline value for infection);

Group 3: Miconazole (2% cream): Estrogenized and infected (inoculated with yeast) treated with 27 µL of 2% miconazole cream (positive control), intravaginally;

Group 4: Vehicle: Estrogenized and infected (inoculated with yeast) treated with a 27 µL mixture of DMA (2.7 µL) + 1% HPMC (24.3 µL), intravaginally;

Group 5: *N*-3mb-bs (12.5 mg/kg): Estrogenized and infected (inoculated with yeast) treated with 27 µL suspension of 0.25 mg *N*-3mb-bs dissolved in DMA (2.7 µL) + 1% HPMC (24.3 µL), intravaginally.

On day 6, mice were euthanized and 100 µL of vaginal lavage fluid was collected and plated on YPD agar plates supplemented with 100 µg/mL ampicillin. After incubation at 30 °C for 48 h, the colonies were counted.

The vaginal tissues were also extracted for histopathological analysis. After being fixed in 10% neutral-buffered formalin, the tissues were dehydrated with a gradual increase in alcohol concentrations, followed by xylene. After being embedded in paraffin, the tissues were cut into 5 μm thick sections and mounted on a poly-l-lysine-coated slide. The tissues were stained with hematoxylin and eosin (H&E). The images were acquired using a Zeiss Axio Scope A1 microscope (Micro-Optics Precision Instruments, Fresh Meadows, NY, USA) with Zeiss Zen 2.3 software.

### 2.6. Immunohistochemistry Analysis

Immunohistochemistry (IHC) was performed as described previously by Brown and colleagues [24]. Tissue sections (5 μm thick) were cut from formalin-fixed, paraffin-embedded vagina samples. The paraffin-embedded vaginal tissues, following hydration, were incubated overnight with antigen retrieval solution. Briefly, tissues were treated with primary antibody myeloperoxidase (1:1000; Cat # ab208670; Abcam, Waltham, MA, USA) in normal goat serum for 2 h at room temperature. After quenching with 0.3% hydrogen peroxide, the slides were washed with 1× TBS and incubated with biotinylated secondary antibody followed by ABC reagent, and finally developed with DAB substrate solution. The tissues were counterstained with methyl green (1% *w*/*w*) followed by dehydration and mounted with coverslips to observe at 200× magnification by light microscopy.

### 2.7. In Vitro Skin Irritation Test

The in vitro skin irritation test was conducted utilizing MatTek Corporation’s Reconstructed Human Epidermal Model EpiDerm™ in accordance with the manufacturer’s instructions, meeting the criteria outlined by the Organization for Economic Co-operation and Development [25]. In brief, applications consisted of either 30 µL of *N*-3mb-bs (at test concentrations of 10, 25, or 50 µM), PBS, or 0.1% DMSO, or 30 µL of 1% Triton-X100 (utilized as a positive control for irritation). Following application, all plates were incubated in a humidified environment for an additional 35 ± 1 min at 37 ± 1 °C, 5 ± 1% CO_2_, and 90 ± 10% relative humidity. Subsequent to the 1 h incubation period, the dosed tissues were removed from the incubator and transferred to a sterile hood. The tissues underwent rinsing 15 times with sterile PBS, followed by incubation of the blotted tissue inserts for 24 ± 2 h. The following day, cell viability was assessed using the MTT assay, as described previously [20,25].

### 2.8. Statistical Analysis

Biological analysis results are expressed as mean ± standard error of the mean (SEM) derived from a minimum of three representative experiments. Data underwent analysis via one-way ANOVA followed by Dunnett’s multiple comparison test using GraphPad Prism 8.0. Statistical significance was defined as *p* < 0.05.

## 3. Results

### 3.1. Chemical Properties and Determination of MIC

The chemical properties of the test compounds were predicted using the SwissADME platform, as shown in Table 1. The water solubility of *N*-allyl-bs and *N*-3mb-bs (202 and 70.2 µg/mL, respectively) were higher than EB (33.0 µg/mL). HPLC results revealed high purity of *N*-allyl-bs and *N*-3mb-bs (Supplementary Material Figure S2).

To confirm the growth-inhibitory effect of EB analogs in the living yeast cells, we determined the minimum inhibitory concentration (MIC) with colorimetric and turbidity assays. The MIC of EB-treated wells in S1 (FLU-sensitive) and S2 (FLU-resistant) strains was 25 µM after 24 h and 48 h incubation in both colorimetric and turbidity assays. The MIC in both colorimetric and turbidity assays of *N*-3mb-bs-treated wells was similar to that of EB (25 µM), with one exception (its MIC value was 50 µM at 24 h in the turbidity assay) (Table 2).

The MIC of EB-treated wells was 25 µM, while those treated with *N*-allyl-bs ranged from 50 to 100 µM. Whereas *N*-3mb-bs proved to be equipotent to EB in both test strains of *C. albicans*, this analog possesses approximately twofold higher water solubility (predicted) compared to EB (Table 1). For FLU-treated wells, the MICs were all >100 µM, confirming its fungistatic property (Supplementary Material Figure S3).

### 3.2. Half Maximal Inhibitory Concentration (IC_50_ Values) in KB-3-1 Cells and C. albicans

The IC_50_ values were determined by MTT assay and turbidity assay. The results are presented in Table 3. The MTT assay was performed on human epidermoid carcinoma cells KB-3-1 for 24 h incubation. Both *N*-allyl-bs and *N*-3mb-bs exhibited more potent antiproliferative activity (IC_50_ = 45.81 and 54.92 µM, respectively) in comparison to EB (89.74 µM). IC_50_ values in *C. albicans* were measured from the plot of A_600nm_ vs. test concentration after a 48 h incubation. EB showed IC_50_ values in S1 and S2 strains of 17.06 and 14.28 µM, respectively. *N*-allyl-bs showed a moderate but lower antifungal activity with IC_50_ values of 29.78 (S1) and 28.05 µM (S2). *N*-3mb-bs was equally potent as EB in S1 with the IC_50_ of 18.21 µM; however, *N*-3mb-bs exhibited approximately threefold stronger antifungal activity than EB in the S2 strain. Note that the log *p* value appeared to correlate with the observed IC_50_ values of the test compounds, with EB and *N*-3mb-bs (log P of ~3 and ~3.3, respectively) showing greater antifungal activity in both test strains than *N*-allyl-bs (log P~2.3).

### 3.3. Effect on Medium Acidification in C. albicans S1

Since Pma1p is critical in regulating intracellular pH and uptake of nutrients [13,27,28], a modified version of the medium acidification assay detecting the effects of Pma1p described by Perlin et al. [27] was performed. Inhibition of Pma1p stops protons from being pumped extracellularly, thereby preventing the acidification of the culture medium. As a previously reported inhibitor of Pma1p [11,13], EB suppressed the medium acidification by *C. albicans* S1 in a concentration-dependent manner (IC50_MA_ of 12.50 µM, Table 4). *N*-allyl-bs and *N*-3mb-bs inhibited medium acidification in *C. albicans* S1 with IC50_MA_ values of 6.42 and 19.61 μM at 30 min, respectively. These results demonstrate the Pma1p-inhibitory effects of *N*-allyl-bs and *N*-3mb-bs. FLU, as the negative control, had no effect on medium acidification (>30 µM) (Table 4).

However, both *N*-allyl-bs and *N*-3mb-bs showed Na^+^, K^+^-ATPase inhibitory activity (purified from pig kidney outer medulla) (Supplementary Material Figure S4) [29,30].

### 3.4. Validation of the Mouse Model of VVC and Intervention Study

#### 3.4.1. Validation

Vaginal lavage samples isolated from mice 6 days post-infection with the S1 strain contained 222 yeast proteins, as determined by proteomics analysis. Table 5 contains a list of the ten most abundant *C. albicans* proteins observed therein. Glycolytic enzymes and virulence factors, including members of the secreted aspartic proteases (SAPs), heat shock protein SSA1, and RBT4 were present. Because uninfected mice showed no yeast proteins in the vaginal lavage fluid at all, a differential analysis between the two groups such as a volcano plot was not possible. However, since the vaginal lavage fluid from uninfected mice did not express any detectable *C. albicans* proteins, the presence of said proteins in the yeast-infected group 6 days after inoculation is confirmatory of a bona fide vaginal yeast infection in these mice.

**Figure 2:**
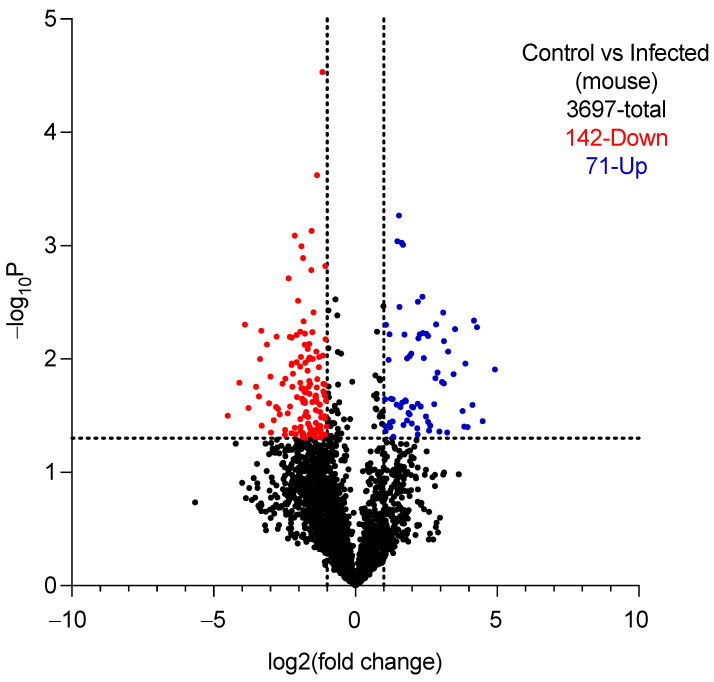
Volcano plot of differentially expressed proteins in naïve uninfected vs. estrogenized/infected BALB/c mice. Black dots are non-differentially expressed proteins while dashed lines indicate the cut-off on each axis.

More than 3500 mouse proteins were observed in the vaginal lavage fluid of both naïve uninfected mice and estrogenized/yeast-infected mice, with 213 proteins differentially expressed between the two groups (Figure 2).

A heat map of the 71 differentially upregulated and the top 100 downregulated proteins is also provided (see Supplementary Material Figure S5). Among the mouse proteins detected in vaginal lavage fluid, the most abundant in estrogenized/infected mice relative to naïve uninfected mice included eosinophil peroxidase, CD109 antigen, desmocollin-1, and gasdermin A (Supplementary Material Table S1) [45,46,47,48,49,50,51,52,53,54].

#### 3.4.2. Efficacy of Intervention Treatments

**Figure 3:**
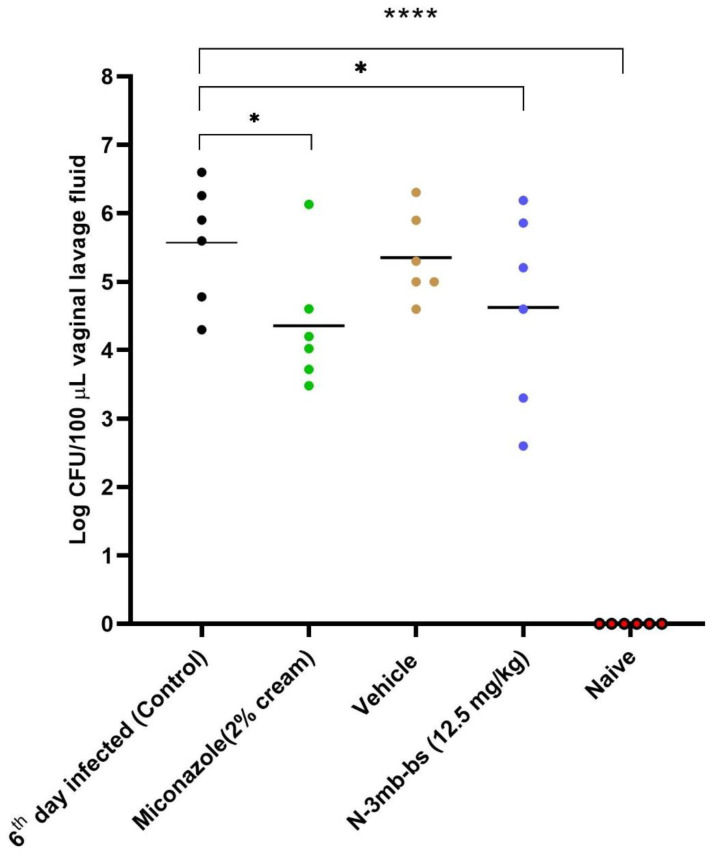
The effectiveness of *N*-3mb-bs (12.5 mg/kg) was evaluated in the mouse model of vulvovaginal candidiasis (VVC). The presented data indicate the colony-forming units (CFUs) obtained from each animal within the respective groups. The fungal burden of murine VVC was assessed using a log10 scale (CFU/100 µL) in the vaginal lavage. Miconazole was employed as the positive control group for treatment. Each point on the curve corresponds to an individual mouse, while the horizontal lines depict the geometric mean of each group. Statistical significance is denoted as * *p* < 0.05 vs. 6th day infected, and **** *p* < 0.0001 vs. 6th day infected.

Based on the in vitro findings, *N*-3mb-bs was chosen for intervention in the mouse model of vulvovaginal candidiasis (VVC) induced by *C. albicans* S1. Mice with VVC received respective treatments via the intravaginal route once daily on days 3, 4, and 5. Following euthanasia, vaginal lavage fluid was collected, and colony-forming units (CFU/100 µL) were enumerated (refer to Supplementary Material Figure S6). Analysis of vaginal fungal burden revealed a high infection rate in the group on the 6th day post-infection. Treatment with *N*-3mb-bs suspension (at a dose of 12.5 mg/kg) resulted in a reduction in fungal burden, with only 11.48% of yeast remaining in the lavage, showcasing efficacy comparable to that of miconazole in reducing *C. albicans* S1 levels (refer to Figure 3 and Table 6).

### 3.5. Histological Analysis of the Vaginal Tissue

**Figure 4:**
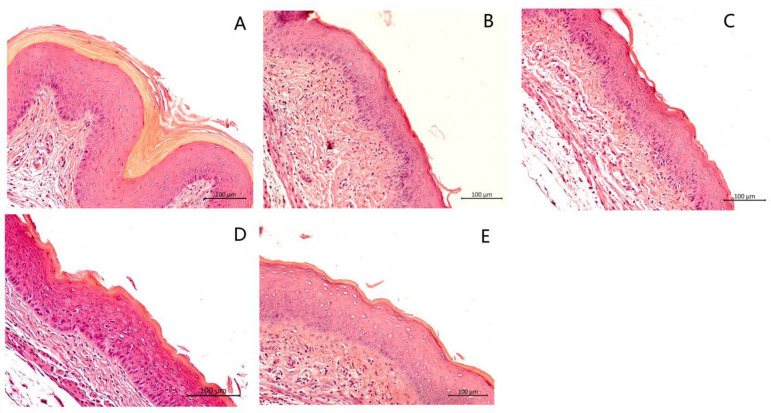
Histopathological analysis of vaginal tissues with H&E staining. Mouse vaginal tissue was excised longitudinally. Each section of paraffin-embedded tissues was stained with H&E and then observed using light microscopy. (**A**) Naive group; (**B**) 6th day infected, untreated; panels (**C**–**E**): 6th day infected and treated with (**C**) vehicle, (**D**) miconazole (2% cream), or (**E**) *N*-3mb-bs (12.5 mg/kg). Magnification: 200×; scale bars: 100 μm.

H&E analysis was carried out to determine inflammation and damage to the vaginal mucosa, such as vaginal epithelial hyperplasia, edema, and damage to the keratin layer. Tissues from the 6th day infected but untreated group (Figure 4B) and vehicle group (Figure 4C) showed edema of the vaginal epithelial cells and a ruffled keratin layer, whereas the tissue sections from the naïve uninfected group (Figure 4A) appeared normal, well defined, and keratinized. Mice treated with *N*-3mb-bs (12.5 mg/kg) (Figure 4E) and miconazole (Figure 4D) showed slight epithelial distress with a stratified and keratinized epithelium, indicating the effective treatment of VVC. No histopathological signs of toxicity were observed in the acute toxicity study (Supplementary Material Figure S7).

### 3.6. Immunohistochemical Analysis

Myeloperoxidase (MPO) is an enzyme that is present in polymorphonuclear immune cells (PMNs) [55]. PMNs provide the first line of defense to the host in the inflammatory responses via mobilization towards the insult sites to kill invading pathogens [56].

**Figure 5:**
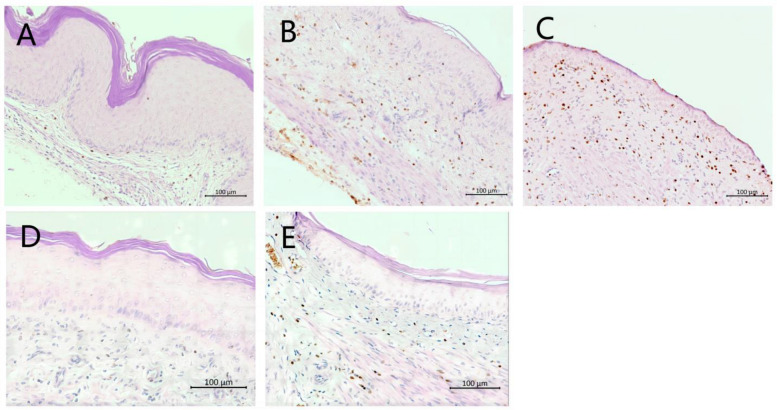
Immunohistochemistry analysis of vaginal tissues with an antibody to MPO. (**A**) Naive group; (**B**) 6th day infected; panels (**C**–**E**): 6th day infected and treated with (**C**) vehicle, (**D**) miconazole (2% cream), or (**E**) *N*-3mb-bs (12.5 mg/kg). Magnification: 200×; scale bars: 100 μm.

**Figure 6:**
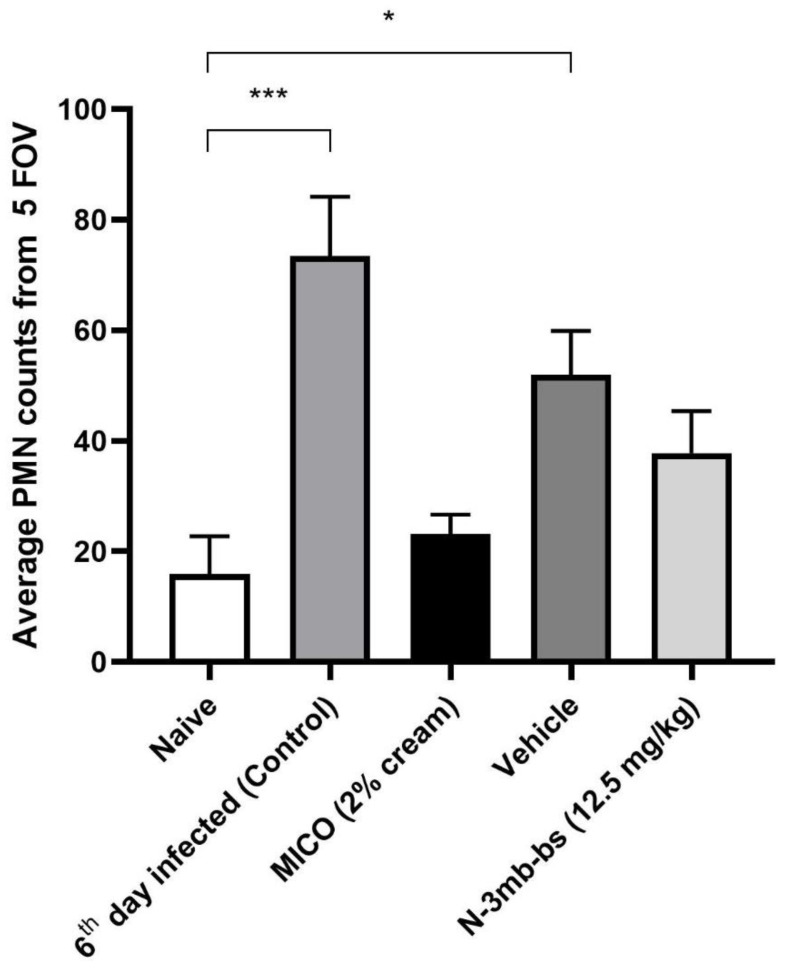
PMN counts of vaginal tissues. Sixth day infected group showed high amount of MPO+ cells in the vaginal mucosa relative to uninfected naïve mice. Mice infected with yeast and treated with *N*-3mb-bs showed fewer PMN counts compared to control (infected but untreated) and vehicle (infected but treated with vehicle only) groups. Each bar represents an average of four tissues per treatment group. Data are expressed as mean ± SEM (n = 4). * *p* < 0.05 vs. naive group. *** *p* < 0.0005 vs. naive group.

IHC analysis of vaginal tissues with an antibody to MPO showed slight presence of MPO in the naïve group (Figure 5A) while showing higher levels of MPO in the 6th day infected group (Figure 5B), confirming the inflammatory response during VVC infection. The *N*-3mb-bs-treated group showed a decrease (Figure 5E) in expression of MPO compared to the 6th day infected (control, Figure 5B) and vehicle group (Figure 5C). The *N*-3mb-bs-treated group had similar MPO expression as that of the miconazole-treated group (Figure 5D). The average number of MPO cells across five randomly chosen fields of view (FOV) for each section was then counted and the average was plotted on a graph (Figure 6). Infected mice treated with *N*-3mb-bs (12.5 mg/kg) or miconazole each showed a similar number of MPO+ cells as naïve, uninfected mice, with less PMNs present compared to vehicle or infected control mice. Its antifungal efficacy was comparable to miconazole in the mouse model of VVC.

### 3.7. Skin Irritation Test

Using reconstructed human epidermis (Mattek Epiderm) and OECD test guideline No. 439 [25], no skin irritation was observed at test concentrations of 10, 25, or 50 µM of 3-mb-bs (Supplementary Material Figure S8).

## 4. Discussion

Vulvovaginal candidiasis (VVC), primarily caused by *C. albicans*, affects millions of women annually [57], presenting a significant public health concern due to its frequent occurrence as an acute inflammatory infection during women’s reproductive years [58]. Despite the availability of azoles as standard antifungal therapy, their efficacy limitations, increasing resistance, and fungistatic properties contribute to recurrent infections and heightened morbidity. Oral fluconazole (FLU) has been widely used over the past two decades for VVC prevention and treatment. However, its extensive use has resulted in the emergence of FLU-resistant *Candida* strains, significantly impeding successful VVC management [59]. Hence, the identification of novel agents effective against these resistant strains is imperative to enhance treatment options and patient outcomes [60]. EB has been shown to possess antifungal activity towards *Candida* species and has been proposed as a potential alternative to traditional antifungal treatments [9,14,61]. However, considering the poor aqueous solubility of EB, novel analogs, *N*-allyl-bs, and *N*-3mb-bs, with improved aqueous solubility based on prediction by the SwissADME platform, were previously developed [18,19,62]. It should be noted that the actual water solubilities of *N*-allyl-bs and *N*-3mb-bs remain unknown at the present time and must be validated experimentally in the future.

*N*-allyl-bs is a smaller structure than EB and possesses an allyl group, which could bind covalently to various intracellular proteins and, therefore, is unlikely to be considered a drug-like compound category. However, it may serve as a precursor molecule that can be further used in pharmacological applications. In addition, the allyl group is a well-known constituent of sulfur and selenium-based components of garlic with proven cancer-preventive properties [63,64]. And while all three test compounds look similar in their 2D structures (Figure 1), they all have distinctive differences when their 3D structures are compared. For example, the isopentyl group of *N*-3mb-bs brings more steric hindrance than the other two, while EB is likely to be more hydrophobic than the other two because of the aromatic phenyl ring, which further allows for it to interact with proteins through a pi–pi interaction.

Another reason that *N*-allyl-BS and *N*-3mb-BS were selected for this study is because they exhibit increased bioactivity among other previously tested EB-like derivatives. To this end, both compounds are active cytotoxic agents that induce oxidative stress and inhibit the growth of DU-145 and PC-3 cancer cell lines [19,65]. During previous investigation of *N*-terpene and *N*-amino acid benzisoselenazolones, we observed that the presence of the 3-methylbutyl chain incorporated in the structure of an *N*-menthyl substituent or attached to the nitrogen atom of a L-leucine derivative could be the reason of their elevated anticancer activity [19,65].

To confirm their growth-inhibitory effect in the living yeast cells, we here determined the minimum inhibitory concentration (MIC) in colorimetric and turbidity assays and proved *N*-allyl-bs and *N*-3mb-bs to be potent antifungal agents in S1 (FLU-sensitive) and S2 (FLU-resistant) strains of *C. albicans*. In our opinion, the observed effect results from the structural combination of the aromatic phenyl ring of the benzisoselenazolone core, often necessary for the proper receptor binding, suitable length, and hindrance of the isopentyl chain, that probably ensures an adequate solubility and bioavailability (*N*-3mb-BS), or a reactive electron rich allyl group that can easily undergo a biochemical transformation (*N*-allyl-BS).

The plasma membrane H^+^-ATPase (Pma1p) is essential for nutrient uptake and stress response in the physiology of fungi [28] and serves as a promising target for antifungal reagents. We carried out medium acidification assays with these two EB analogs and observed that they both exerted an inhibitory action on H^+^-ATPase pump; however, *N*-allyl-bs was found to be more potent than either EB or *N*-3mb-bs. It is conceivable that the higher concentration of *N*-3mb-bs required for Pma1p inhibition relative to the *N*-allyl-bs analog is related to the experimental conditions used here. Thus, the acidification assays, which progress over a period of 30 min but are performed only after a preliminary incubation of 15 min using intact yeast cells, may require different pre-incubation times with different analogs of EB (i.e., the diffusion of bulkier *N*-3mb-bs into the cell might be slower than that of *N*-allyl-bs/or it might interact with key cysteines of Pma1p with a different rate). These latter possibilities are worthy of future study. Moreover, it should also be pointed out that the antifungal MIC assays are carried out for 24 or 48 h while the medium acidification assays are carried out for 30 min. Thus, future studies should describe inter-relationships between MIC and IC_50MA_ values, perhaps by adjusting the time courses.

Turning to the mouse model of VVC, here, we used the clinical isolate S1 of *C. albicans* [66], which is described extensively in our previous works [13,15,26], and found that mice intravaginally inoculated with this strain expressed yeast proteins 6 days later in the vaginal lavage fluid including glycolytic enzymes and virulence factors, including members of the secreted aspartic proteases (SAPs), heat shock protein SSA1, and RBT4. This demonstrates that the S1 strain establishes a vaginal yeast infection with virulence factors typically observed in systemic infections [35,36,37,38,39,40,41,42,43,44]. Among the mouse proteins discovered in the vaginal lavage fluid, 213 proteins were differentially expressed between the two groups. Since the focus of the present study was to use the mouse VVC model to determine the extent to which *N*-3mb-bs reduces fungal burden and the presence of infiltrating immune cells in the vaginal mucosa of S1-infected mice, the vaginal lavage proteomics data reported here confirms that the use of S1 strain, a clinical isolate of *C. albicans*, to infect the mice was effective. However, much more work is required to decipher the extent to which *N*-3mb-bs alters the proteins in the vaginal lavage fluid and future work will aim to understand this aspect.

We conducted the in vivo study of *N*-3mb-bs in the mouse model of VVC using *C. albicans* S1 to infect the mice. After 3 days of treatment (12.5 mg/kg), the *N*-3mb-bs suspension significantly reduced the fungal burden, with only 11.48% of yeast remaining in the lavage compared to infected control mice (Figure 3, Table 6). H&E analysis showed that mice treated with *N*-3mb-bs (12.5 mg/kg) exhibited reduced epithelial distress and a keratinized epithelium. Immunohistochemical analysis showed that 3-mb-bs reduced the MPO expression in vaginal tissues from infected mice, indicating a dramatically reduced inflammatory response with less PMNs presence.

Some issues in this study need further consideration. Although *N*-allyl-bs and *N*-3mb-bs showed suppression effects on Pma1p, these compounds also inhibited Na^+^, K^+^-ATPase, an enzyme critical for animal cells (host) homeostasis. The inhibiting effect observed in the micromolar range (Supplementary Material Figure S4) was practically irreversible but prevented by the presence of GSH in physiological intracellular concentrations. Thus, the reactivity towards SH groups is a significant obstacle for the systemic administration of ebselen and its analogs and surely contributes to the molecular basis of the chronic toxicity of organoselenium compounds [67]. On the other hand, when used acutely and in the vaginal milieu, the use of organoselenium compounds as inhibitors of specific thiol-containing enzymes such as the yeast Pma1p appears to be safe [15,26] and may be of therapeutic significance. In this study, upon intravaginal administration as a suspension, *N*-3mb-bs demonstrated notable antifungal activity without any observable adverse effects on the overall health of the mice. For instance, the mice maintained responsiveness, remained active, and did not exhibit weight loss throughout the duration of the study. No histopathological signs of toxicity of *N*-3mb-bs were observed, indicating the safety of this compound. Using reconstructed human epidermis (Mattek Epiderm) and OECD test protocol No. 439 [25], no skin irritation was observed at test concentrations of *N*-3mb-bs up to 50 µM (Supplementary Material Figure S8). This indicates that *N*-3mb-bs should be considered for future study as an antifungal with potential application to humans. Therefore, further research is needed to establish the safety and efficacy of *N*-3mb-bs as a biological material for treatment of candidiasis, as well as to develop methods for its large-scale production and potential development into useful antifungal materials.

Only the FLU-sensitive S1 strain was used here to establish VVC infection in the mouse model. Future work will be required to determine the extent to which 3-mb-bs inhibits VVC infection by FLU-resistant strains of yeast such as S2 and others.

## 5. Conclusions

Our study showed the following:

- *N*-3mb-bs inhibits the growth of S1 (FLU-sensitive) and S2 (FLU-resistant) strains of *C. albicans* in vitro (strength);
- *N*-3mb-bs inhibits Pma1p activity in the *C. albicans* test strain S1 in vitro (strength);
- *N*-3mb-bs inhibits the Na^+^, K^+^-ATPase obtained from pig (weakness, suggests “off-target” effects may occur in humans);
- *C. albicans* clinical isolate S1 is an effective inducer of infection and causes a robust vaginal yeast infection in the mouse model of VVC (strength);
- *N*-3mb-bs reduces vaginal colonization by S1 and relieves inflammation and damage to the vaginal mucosa (strength);
- *N*-3mb-bs reduces MPO expression and PMN infiltration in the infected vaginal tissues (strength).

Overall, our study presents *N*-3mb-bs, a *N*-substituted-benzisoselenazol-(2H)-one, as a promising drug candidate for the treatment of VVC and suggests that it may have potential for use as an antifungal biomaterial (e.g., as a wet wipe to sterilize surgical equipment during surgery in immunocompromised patients).

## Figures and Tables

**Table 1:** Molecular properties of *N*-allyl-bs, *N*-3mb-bs, and EB ^a^.

Compounds	Molecular Weight (g/mol)	Water Solubility (µg/mL)
*N*-allyl-bs	238.14	202
*N*-3mb-bs	268.21	70.2
EB	274.18	33.0

^a^ http://www.swissadme.ch/, accessed on 3 November 2023.

**Table 2:** Growth inhibitory effects in S1 and S2 strains of *C. albicans* in different assays *.

Compounds			*N*-allyl-bs	*N*-3mb-bs	EB **	FLU **
Colorimetric assay	*C. albicans* S1 (µM)	24 h	50	25	25	>100
		48 h	100	25	25	>100
	*C. albicans* S2 (µM)	24 h	100	25	25	>100
		48 h	50	25	25	>100
Turbidity assay	*C. albicans* S1 (µM)	24 h	50	25	25	25
		48 h	50	25	25	25
	*C. albicans* S2 (µM)	24 h	50	50	25	>100
		48 h	50	25	25	>100

* In the colorimetric assay, the MIC for FLU was >100 µM in both strains. ** Previously reported [26].

**Table 3:** Effects of *N*-allyl-bs and *N*-3mb-bs on cell viability.

Compounds	Log P ^#^	MTT Assay IC_50_ (µM) KB-3-1 Cells	Turbidity Assay IC_50_ (µM)	
			*C. albicans* S1	*C. albicans* S2
*N*-allyl-bs	2.29	45.81 ± 4.60	29.78 ± 0.13	28.05 ± 0.47
*N*-3mb-bs	3.29	54.92 ± 7.00	18.21 ± 0.05	5.35 ± 0.07
EB	2.92	89.74 ± 3.42 **	17.06 ± 1.01 **	14.28 ± 0.46 **
FLU	−0.12	>1000 **	6.01 ± 0.10 **	>100 **

^#^ Prediction from Molinspiration Property Calculation Service (www.molinspiration.com, accessed on 20 December 2023). ** Previously published [26].

**Table 4:** Inhibition of Pma1p by *N*-allyl-bs and *N*-3mb-bs in *C. albicans* S1 ^a^.

Compounds	IC50_MA_, µM
*N*-allyl-bs	6.42 ± 0.81
*N*-3mb-bs	19.61 ± 1.29
EB ^b^	12.50 ± 1.10
FLU ^b^	>30

^a^ Values are reported as mean of three experiments ± standard error of the mean (SEM). ^b^ IC50_MA_ values for EB and FLU were published previously [26].

**Table 5:** Most abundant yeast proteins detected in vaginal lavage fluid from S1-infected BALB/c mice.

		Spectral Counts (DDM)		
UniProt ID	Name	Uninfected Mice N = 4 Mean ± SD, [95% CI]	Infected Mice N = 4 Mean ± SD, [95% CI]	Function
A0A1D8PP43	Aldehyde dehydrogenase (Adh1p)	ND *	34.50 ± 14.55 [11.35–57.65]	Oxidoreductase, found in yeast and hyphal forms of *C. albicans* [31].
P46273	Phosphoglycerate kinase (Pgk)	ND	34.25 ± 15.65 [9.34–59.15]	Glycolytic enzyme, found in the cell wall of *C. albicans* [32].
P30575	Enolase 1	ND	31.00 ± 11.17 [13.23–48.77]	Glycolytic enzyme, found in the cell wall of *C. albicans* [33].
Q5ADM7	Glyceraldehyde-3-phosphate dehydrogenase	ND	23.00 ± 17.38 [−4.65–50.65]	Glycolytic enzyme, found in the cell wall of *C. albicans* [34].
P43094	Candidapepsin-5	ND	21.50 ± 9.47 [6.43–36.57]	Secreted aspartic protease involved in the virulence of *C. albicans* [35,36].
Q9URB4	Fructose-bisphosphate aldolase	ND	21.00 ± 10.23 [4.72–37.28]	Glycolytic enzyme, antibodies to which are found in sera of patients with candidemia [37,38].
P41797	Heat shock protein SSA1	ND	17.50 ± 10.47 [0.84–34.16]	Member of Hsp70 family, involved In virulence of *C. albicans* [39,40].
Q5A8N2	Candidapepsin-4	ND	16.50 ± 10.47 [−0.16–33.16]	Secreted aspartic protease involved in the virulence of *C. albicans* [41,42].
Q5AC08	Candidapepsin-6	ND	12.00 ± 5.60 [3.09–20.91]	Secreted aspartic protease involved in the virulence of *C. albicans* [41,43].
Q5AB48	RBT4	ND	10.00 ± 4.24 [3.25–16.75]	Secreted protein that acts as a virulence factor during infections [43,44].

* ND: not detected.

**Table 6:** Growth inhibition of *C. albicans* S1 strain in the VVC mouse model.

Groups	Log CFU/100 μL	Remaining *C. albicans* S1 Compared to Control (100%)
6th day infected (control)	5.57	100
Miconazole	4.36	6.17
Vehicle	5.35	60.26
*N*-3mb-bs	4.63	11.48
Naive	-	-

## Data Availability

Data are contained within the article and Supplementary Materials. Raw mass spectrometry data are available at University of California San Diego MassIVE (Dataset MSV000093920). All other data are contained within the article.

## Supplementary Materials

The following supporting information can be downloaded at: https://www.mdpi.com/article/10.3390/cimb46030157/s1, Figure S1. Study design for intervention treatment of murine VVC; Figure S2. HPLC analysis and chromatographic separation of *N*-allyl-bs and *N*-3mb-bs; Figure S3. Representative image of colorimetric assay in different strains of *C. albicans*; Figure S4. Na^+^, K^+^-ATPase activity; Figure S5. Heat map of counts from differential regulated mouse proteins in infected mice relative to naïve, uninfected mice; Table S1. Most abundant mouse proteins detected in the vaginal lavage fluid of S1-infected BALB/c mice; Figure S6. Representative image of fungal burden. On days 3, 4, and 5, mice with VVC received respective treatments once per day; Figure S7. Acute toxicity analysis of *N*-3mb-bs was performed as indicated in Figure S1 without the inoculation of *C. albicans* S1 on day 0; Figure S8: Skin irritation test of *N*-3mb-bs in Mattek EpiDerm^TM^ reconstructed human epidermal model.
