# Supplementary material for: N-3-Methylbutyl-benzisoselenazol-3(2H)-one Exerts Antifungal Activity In Vitro and in a Mouse Model of Vulvovaginal Candidiasis
Source: Curr Issues Mol Biol. 2024 Mar 14;46(3):2480–96. doi: 10.3390/cimb46030157 (PMC10969377; doi:10.3390/cimb46030157)
Supplement: Supplementary file 1 [file cimb-46-00157-s001.zip › cimb-2839800-supplementary.pdf]

Supplementary Materials

N-3-Methylbutyl-Benzisoselenazol-3(2H)-One Exerts Antifungal Activity *In Vitro* and in a Mouse Model of Vulvovaginal Candidiasis

SUPPLEMENTARY DATA

Figure S1 Study design for intervention treatment of murine VVC

In brief, female BALB/c mice weighing between 18 and 22 g were procured from Taconic Laboratories, Inc. (Albany, NY, USA). These mice were housed in an Animal Care Center (ACC) at St. John's University (Jamaica, NY, USA), which is accredited by the Association for Assessment and Accreditation of Laboratory Animal Care (AAALAC). The study was approved by the Institutional Animal Care and Use Committee (IACUC) of St. John's University (Protocol #2003).

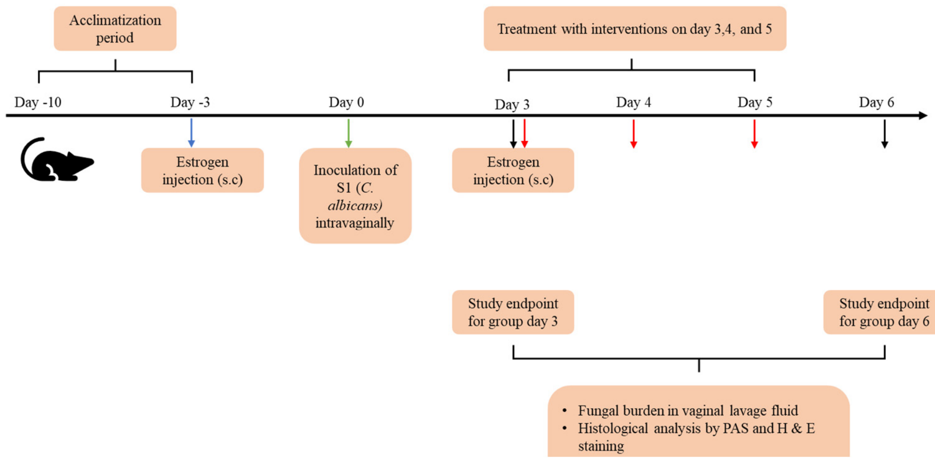

Figure S1 Animals were acclimatized from Day -10 to Day -3 and were administered with s.c. injection of estradiol valerate except for the naive group on day -3. On Day 0, animals were intravaginally inoculated with *C. albicans* S1 strain (5.5 x 10^5 CFU/20 µL); On Day 3, mice received a second subcutaneous injection of estradiol valerate except for the naive group and mice from the Day 3 group which were euthanized. Day 3 also marks the start of the intervention treatments of the respective treatment groups (via intravaginal delivery) and was continued until Day 5. On Day 6, the remaining mice were euthanized, the vaginal lavage fluid was collected, and the vaginal tissues were excised for further studies.

Figure S2 HPLC analysis

Chromatographic separation of N-allyl-bs, and N-3mb-bs was performed using Waters alliance® HPLC equipped with 2998 Photodiode Array (PDA) detector and Hypersil® ODS C18 column (250 mm × 4.6 mm, 5 µm). An optimum isocratic method with a mobile phase ratio of 60:40 Acetonitrile (ACN): HPLC grade water was used. Injection volume and flow rate were set to 10 µL and 1 ml/min respectively. The column temperature was maintained at 25°C. Samples were injected and analyzed using an autosampler and output signal was detected using Empower 3 software at 265 nm. Retention time of N-allyl-bs was 2.05 ± 0.50 min and retention time of N-3mb-bs was 4.44 ± 0.44 min.

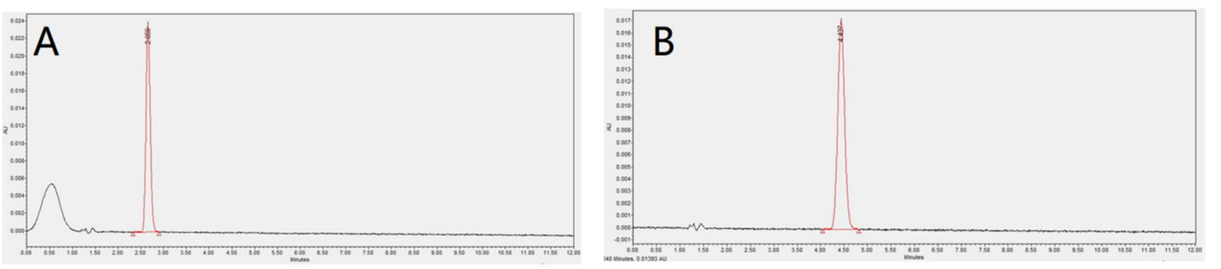

Figure S2 HPLC analysis. (A) N-allyl-bs; (B) N-3mb-bs.

Figure S3 Representative images of colorimetric assays

The yeast suspension was adjusted to achieve an initial inoculum with an A600 of 0.010 in RPMI 1640 medium. In 96-well plates, each well was filled with 100 µL of the yeast suspension followed by 100 µL of the appropriate test compound solutions. All plates were then incubated at 30°C. Following incubation, 20 µL of resazurin dye (0.02 % w/v) was added to each well, and changes in dye color were observed to determine cell viability. MIC values were recorded at 24 and 48 hours (MIC24h and MIC48h, respectively).

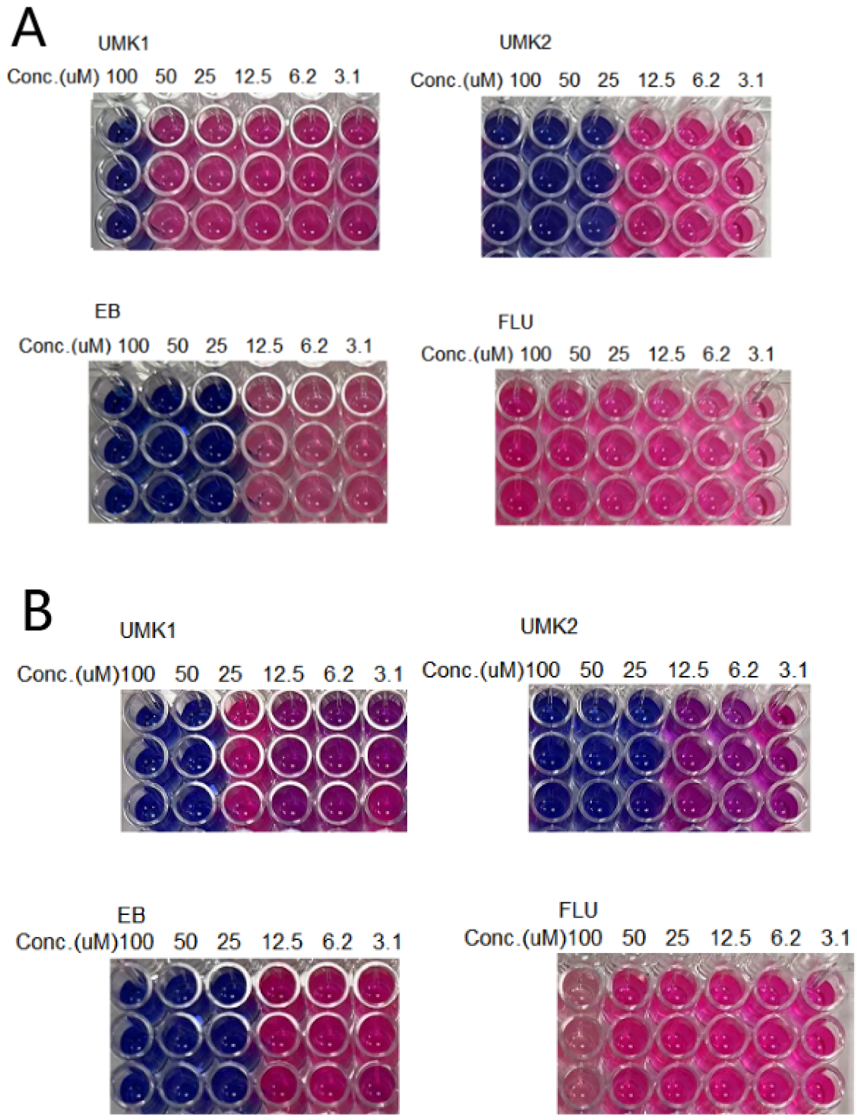

Figure S3. Representative image of colorimetric assay in different strains of *C. albicans*. (A) MIC determination in *C.albicans* S1 after 48 h; (B) MIC determination in *C. albicans* S2 after 48 h. UMK1 is *N*-allyl-bs and UMK2 is *N*-3mb-bs.

Figure S4 Na^{+}, K^{+}-ATPase activity

Methods:

Na+,K+-ATPase was purified from pig kidney outer medulla, as previously described [29]. The interactions with the inhibitors were estimated from the decrease of the ouabain-sensitive hydrolytic activity. The assays were carried out at 37 °C in a medium consisting of 130 mM NaCl, 20 mM KCl, 4 mM $MgCl_2$ and 20 mM histidine (pH 7.4). The enzyme was pre-incubated with the compounds for 1 hour at 37 °C prior to addition of 3 mM ATP, whereupon hydrolysis of ATP was allowed to proceed for 2 min. In the time-dependent batch experiments, i.e. binding of the compounds or re-activation by 5 mM GSH, the enzyme samples were taken after different periods of time and investigated for the residual activity by measuring ATP hydrolysis for the same 2 min. Specific Na+,K+-ATPase activity was calculated as difference in P_i release in the absence and presence of 1 mM ouabain [30]. The reaction with the test compounds is irreversible, as illustrated by Figure S4, Panel C, where the enzyme activity is not restored by addition of 5 mM GSH after complete inactivation is reached.

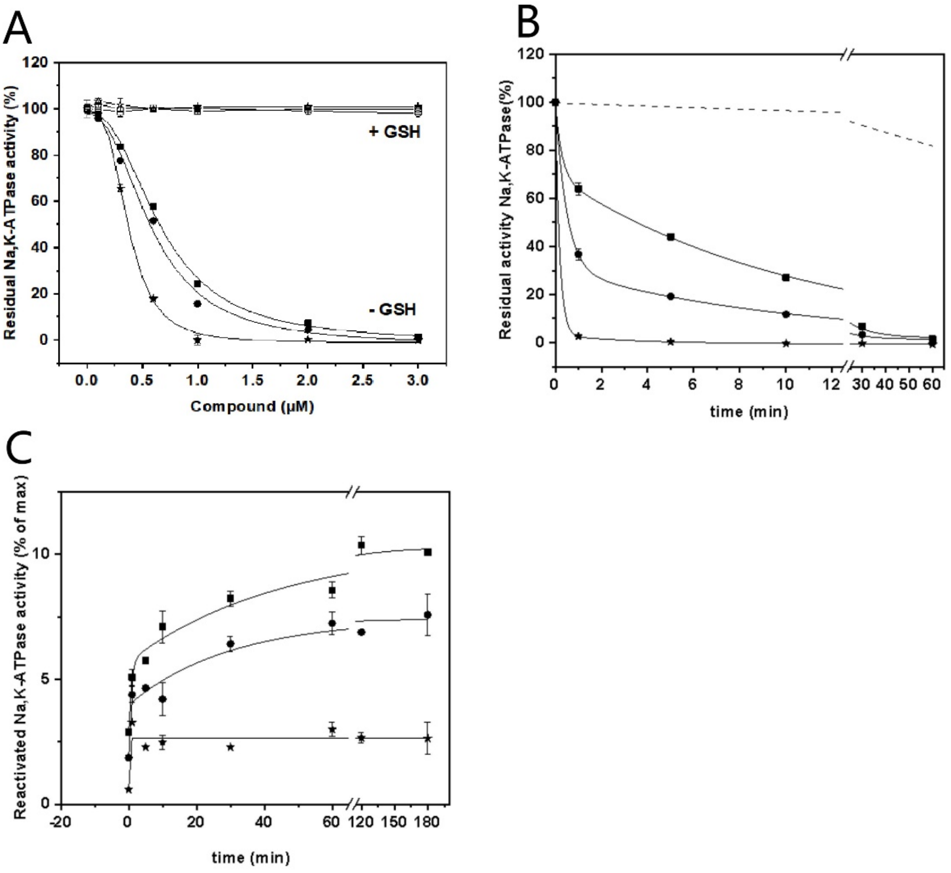

Figure S4 Effect of test compounds in the Na^+, K^+-ATPase activity. (A) Inactivation of the Na^+, K^+-ATPase by ebselen and its analogs in the absence and presence of 5 mM GSH. (B) Time course of inactivation of the Na^+, K^+-ATPase by 3 μM ebselen or its analogs. Non-specific inhibition by DMSO is shown as a broken line. (C) Time course of re-activation of the Na^+, K^+-ATPase by addition of 5 mM GSH. The enzyme was initially exposed to 3 μM inhibitors for 1 hour. N-allyl-bs (squares), N-3mb-bs (circles), ebselen (stars). The data are averages of three independent experiments with the error whiskers representing SD.

Figure S5 Heat map

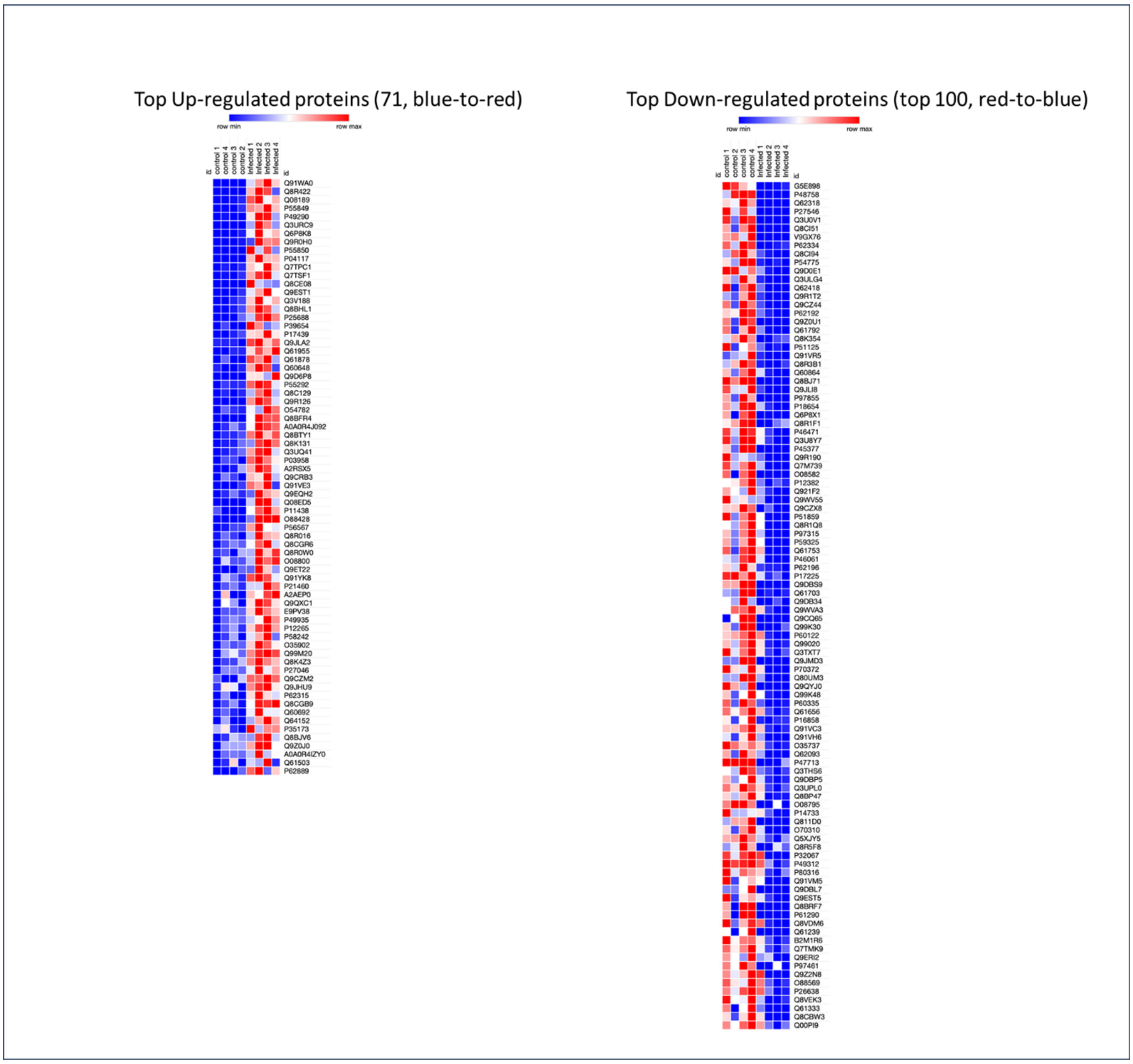

Figure S5 Heat map of counts from differential regulated mouse proteins in infected mice relative to naïve, uninfected mice

Table S1

Most abundant mouse proteins detected in the vaginal lavage fluid of S1-infected BALB/c mice.

UniProt ID	Name	Log2 (Fold Change)	p value	Function
P49290	Eosinophil peroxidase	5.860	0.027	Mediates posttranslational tyrosine nitration of eosinophil granule toxins [45].
Q8R422	CD109 antigen	5.805	0.038	Regulates differentiation of keratinocytes [46].
Q9R0H0	Peroxisomal acyl-coenzyme A oxidase 1	4.910	0.035	Mediates rate-limiting step of peroxisomal beta-oxidation of straight-chain saturated and unsaturated very-long-chain fatty acids [47].
Q6P8K8	Carboxypeptidase A4	4.421	0.010	Regulates cardiac hypertrophy by activating the PI3K-AKT-mTOR signaling [48].
P55849	Desmocollin-1	4.376	0.004	Helps to maintain structural integrity of the skin [49].
Q08189	Protein-glutamine gamma-glutamyltransferase E	4.310	0.006	Involved in the cross-linking of structural proteins to form the cornified envelope in the epidermis [50].
P14901	Heme oxygenase 1	4.289	0.031	Accelerates cutaneous wound healing in mice [51].
P39654	Polyunsaturated fatty acid lipoxygenase ALOX15	4.081	0.039	Catalyzes the stereo-specific peroxidation of free and esterified polyunsaturated fatty acids and generates a host of bioactive lipid mediators [52].
Q9EST1	Gasdermin-A	3.909	0.015	Pore-forming protein expressed in skin that causes membrane permeabilization and pyroptosis [53].
Q61878	Bone marrow proteoglycan	3.897	0.024	Cleaved into eosinophil granule major basic protein (EMBP; MBP) [54].

Figure S6

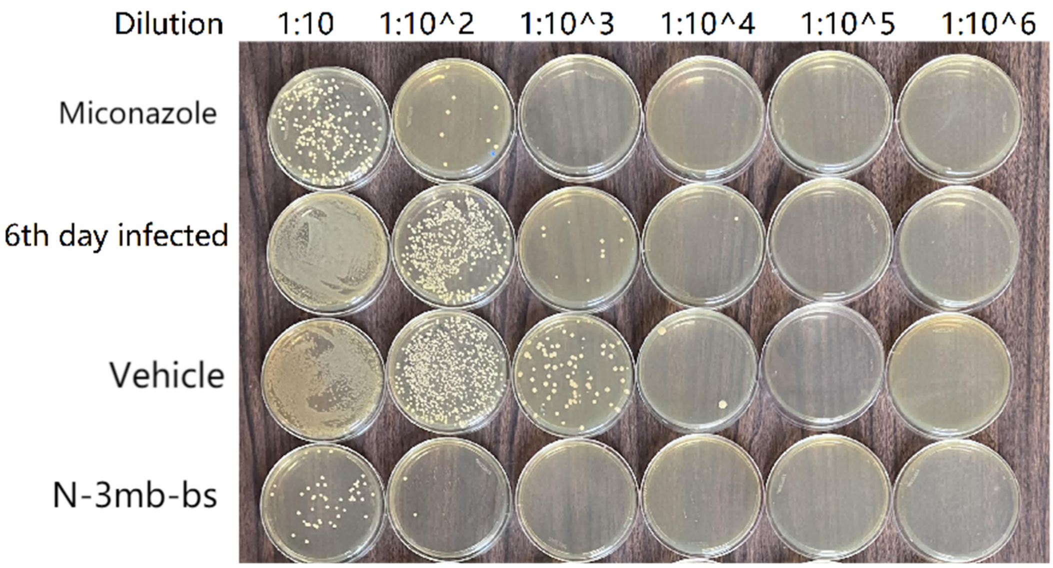

Figure S6 Representative image of fungal burden. On days 3, 4 and 5, mice with VVC received respective treatments once per day. The vaginal lavage fluid was collected, followed by 1:10 serial dilutions and plated on the agar plates with ampicillin (0.1 mg/ml) for 48 h incubation to determine the colony forming units (CFU).

Figure S7

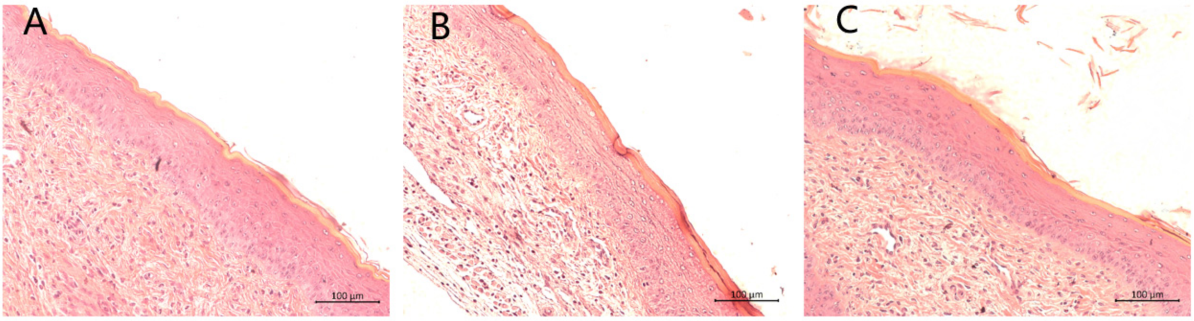

Figure S7 Acute toxicity analysis of *N*-3mb-bs was performed as indicated in Figure S1 without the inoculation of *C.albicans* S1 on day 0. Each section of paraffin-embedded tissues was stained with H & E and then observed using light microscopy. (A) PBS treatment; (B) Vehicle treatment; (C) *N*-3mb-bs (12.5 mg/kg) treatment. Magnification: 200 ×; Scale bars: 100 μm.

Figure S8

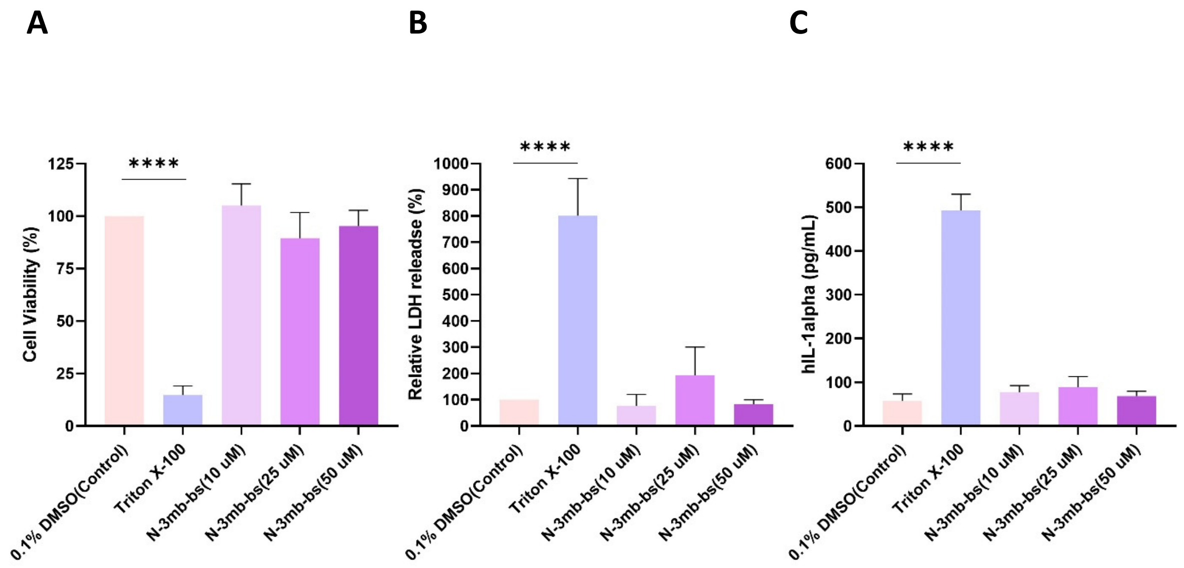

Figure S8: Skin irritation test of *N*-3mb-bs in Mattek EpiDerm^{TM} reconstructed human epidermal model. (A) Cell viability; (B) relative LDH release into tissue culture medium; (C) Level of human IL-1 alpha in the supernatant of culture media. The data were analyzed by one-way ANOVA. ****$p<0.0001$.
